# Characteristics of Novel Anticoagulants versus Vitamin K Antagonists in the Ventricular Mural Thrombus

**DOI:** 10.31083/j.rcm2403074

**Published:** 2023-03-02

**Authors:** Qing Yang, Yan Liang, Xin Quan, Xinyue Lang, Dongfang Gao

**Affiliations:** ^1^National Clinical Research Center of Cardiovascular Diseases, Fuwai Hospital, National Center for Cardiovascular Diseases, Chinese Academy of Medical Sciences and Peking Union Medical College, 100037 Beijing, China; ^2^Emergency Center, Fuwai Hospital, National Center for Cardiovascular Diseases, Chinese Academy of Medical Sciences and Peking Union Medical College, 100037 Beijing, China; ^3^Echocardiographic Imaging Center, Fuwai Hospital, National Center for Cardiovascular Diseases, Chinese Academy of Medical Sciences and Peking Union Medical College, 100037 Beijing, China; ^4^Medical Research & Biometrics Center, National Center for Cardiovascular Diseases, Chinese Academy of Medical Sciences, 102300 Beijing, China

**Keywords:** ventricular mural thrombus, anticoagulants, non-vitamin K antagonist oral anticoagulants, warfarin

## Abstract

**Background:**

To describe the characteristics, treatment practices, and clinical outcomes of patients with ventricular mural thrombus (VMT), with emphasis on the comparison of non-vitamin K antagonist oral anticoagulants (NOACs) and vitamin K antagonists (VKAs).

**Methods:**

We performed a retrospective cohort study between 2010 and 2019 in Fuwai Hospital, China. Patients with VMT newly treated with either NOACs or VKAs were included. The primary outcome was the incidence rate of thrombus resolution at 3 months.

**Results:**

We included 196 patients in total—68.9% (n = 135) were treated with VKAs while 31.1% (n = 61) were on NOACs. Patients with a medical history of heart failure (HF) (odds ratio (OR) 2.10, 95% confidence interval (CI) 1.17 to 3.77, *p* = 0.013) and a lower left ventricular ejection fraction (OR 0.36, 95% CI 0.20 to 0.65, *p* = 0.001) had a higher thrombus resolution. At 3 months, a significant difference was observed in the thrombus resolution between the NOACs and VKAs group with or without adjustment (OR 2.61, 95% CI 1.39 to 4.89, *p* = 0.003; adjusted OR 2.93, 95% CI 1.51 to 5.66, *p* = 0.001). Further investigation revealed that in the majority of the subgroups, individuals receiving NOAC therapy had a superior thrombus resolution than those receiving VKA therapy.

**Conclusions:**

Patients with a medical history of HF or left ventricular ejection fraction <30% experienced greater effectiveness in thrombus resolution. Additionally, the resolution of VMT with NOAC treatment was considerably higher than that with VKA therapy at 3 months, with or without adjusting for baseline variables.

**Clinical Trial Registration:**

This study was registered at ClinicalTrials.gov as NCT 05006677 on August 4th, 2021.

## 1. Introduction

Patients with heart failure (HF) or myocardial infarction (MI) predispose to ventricular mural thrombus (VMT) formation, experiencing a combination of hypercoagulability, abnormal blood flow, and endothelial injury [1, 2]. The most severe VMT complication, which carries a substantial risk of mortality and morbidity, is the incidence of thromboembolism [3]. Whereas the typical use of Vitamin K antagonists (VKAs) for anticoagulation has been largely embraced in clinics, no particular guidelines are provided for the management of VMT [4, 5]. However, due to the drawbacks of warfarin’s late onset, multiple food or drug interactions, and restricted therapeutic window, treatment compliance among patients is relatively low, which increases the likelihood of bleeding or embolism events [6]. Therefore, non-vitamin K antagonist oral anticoagulants (NOACs) are increasingly used as off-label anticoagulant treatments in patients with VMT. From MI and stroke guidelines, the usage of NOACs for the treatment of left VMT was uncertain [7, 8]. Apixaban or rivaroxaban demonstrated a better or comparable thrombus resolution than warfarin in patients with left VMT, and the risks of major cardiovascular adverse events were comparable, according to two prospective multicenter randomized trials [9, 10]. In the No-LVT trial, for example, rivaroxaban was non-inferior to warfarin in terms of thrombus resolution (71.79% vs 47.50%) [10]. Several retrospective studies and meta-analyses also reported that NOACs were non-inferior even superior to warfarin in thrombus resolution while the risk of bleeding or stroke had not reached consensus [11, 12, 13, 14]. Therefore, we aimed to evaluate the characteristics and clinical outcomes of patients with VMT treated with various oral anticoagulants, as well as to explore potential factors related to thrombus resolution.

## 2. Methods

### 2.1 Patient Population

This retrospective cohort study was conducted from July 2010 through October 2019 using electronic medical records of Fuwai Hospital, National Center of Cardiovascular Diseases in China, which was registered in ClinicalTrials.gov: NCT 05006677. The inclusion criteria were: (1) Aged over eighteen years, regardless of sex or occupation; (2) Patients were given a new prescription for a NOAC or a VKA for less than 1 month; (3) VMT was identified newly within 3 months given that mechanized or calcified thrombus was less likely to resolve. Patients who switched medications or discontinued NOACs or VKAs over the course of treatment were excluded, as evidenced by objective data such as prescriptions from cardiologists and oral reports during the interviews. All medications according to the recommendation of guidelines for the treatment of underlying diseases were encouraged.

### 2.2 Definitions

The diagnosis of VMT was confirmed by transthoracic echocardiography, computer tomography (CT), or cardiac magnetic resonance (CMR) imaging. X.Q. and other experts would analyze the image and make a determination. VMT was defined as an abnormal echo mass in the ventricular cavity whose margin was distinct from the ventricular endocardium [15]. Multiple sections confirmed the existence of the thrombus.

The primary outcome was the rate of thrombus resolution determined by repeat imaging within 3 months, and the secondary outcomes included thromboembolism events, bleeding, and all-cause death within 3-month follow-up. We confirmed the resolved thrombus by screening the image data in the electronic system and conducted a survey by phone or media contact with patients to obtain long-term outcomes. Thromboembolism events were defined as the combination of an acute embolism in a coronary or peripheral artery, ischemic stroke, and transient ischemic attack. Bleeding events were classified as major bleeding as defined by the International Society on Thrombosis and Haemostasis [16], clinically relevant non-major bleeding [17], and minor bleeding that failed to comply with the criteria for the abovementioned two categories of bleeding.

### 2.3 Data Collection and Analysis

Data regarding patient demographics (age, gender), clinical characteristics (presenting diagnosis, medical history, and laboratory testing), imaging parameters (left ventricular ejection fraction (LVEF), thrombi features), treatment (type of anticoagulation and combined medications), and clinical outcomes (thrombus resolution, thromboembolism events, bleeding, and all-cause death) were collected. To assure coherence, two colleagues (Q.Y. and X.Q.) separately extracted the data and compared the results. A third researcher then addressed any inconsistencies. Data were obtained from electronic medical records and oral consent was acquired at the time of the telephone interview.

### 2.4 Statistics Analysis

Normally distributed continuous data were presented as mean and standard deviation (SD) while non-normally distributed continuous data by the median and interquartile range (IQR), and the dichotomous data were computed using frequency and percentage [18]. Analysis of variance was used to compare normally continuous variables and the Kruskal-Wallis H test was to compare non-normally distributed continuous variables. When comparing categorical data, the Fisher’s exact test and Pearson chi-squared test (when more than 20% of cells have expected frequencies <5) were applied. Odds ratio (OR) and confidence interval (CI) were estimated with or without adjustment for covariates using Logistic regression models. We split interested characteristics into subgroups to investigate the potential influences on the resolution of the thrombus (e.g., age, gender, body mass index (BMI), LVEF, presenting diagnosis, location of thrombi, medical history, and combination therapy). Subgroup analyses were performed using stratified chi-square models and interactions between subgroups were analyzed using likelihood ratio tests. Two models were taken into account: (i) the unadjusted model (model 1), which contained the main predictor; (ii) model 2, which additionally included LVEF values and medical history of HF. A forest plot was created to display both multivariable Logistic regression and subgroup analysis. The cumulative event probability of resolution was estimated using the Kaplan-Meier method. In addition, a restricted cubic spline curve was used between the continuous variables and the rate of thrombus resolution. Comparisons were regarded as two-sided, and statistical significance was determined by the *p* value of 0.05. All analyses were scheduled for completion with R version 3.5.1 (The R Project for Statistical Computing, Vienna, Austria).

## 3. Results

### 3.1 Patients Characteristics

**Fig. 1.**
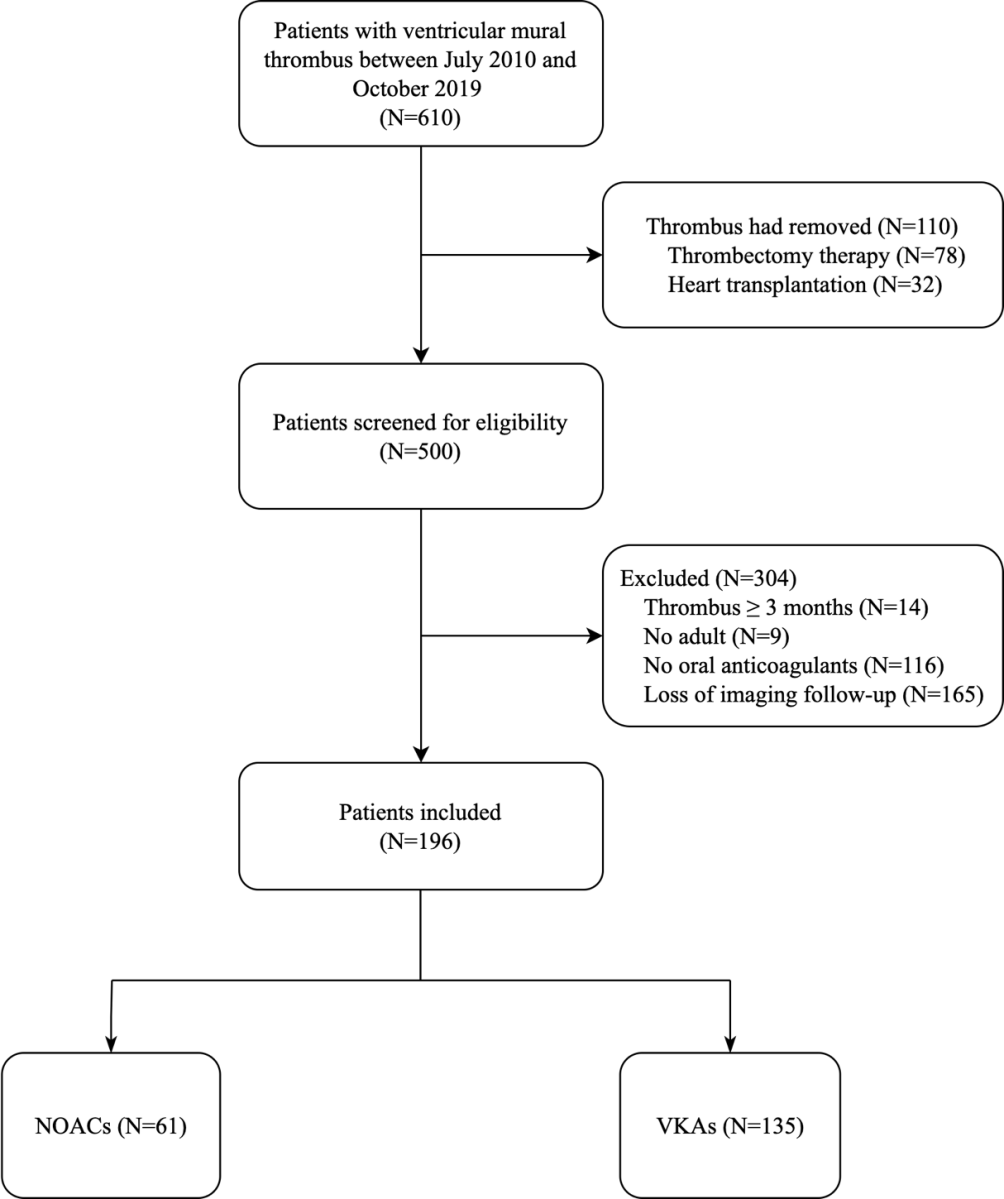
**Flow diagram to show the inclusion and exclusion criteria**. 610 patients were found with ventricular mural thrombus and 196 were included in our analysis—61 received NOACs and 135 received VKAs. NOACs, non-vitamin K antagonist oral anticoagulants; VKAs, vitamin K antagonists.

We identified 610 patients with VMT on the whole between July 2010 and October 2019 throughout this center. There were 78 patients that received thrombectomy therapy or ventricular aneurysm resection while 32 patients with heart transplantation within a 6-week follow-up. Additionally, we disqualified 14 patients with a long history of VMT (more than 3 months) and 9 patients who were not adolescents. Furthermore, 116 patients without oral anticoagulants and 165 patients lost to imaging follow-up were excluded (Fig. 1). Consequently, we enrolled 196 eligible patients: of them, 68.9% (n = 135) received VKAs while 31.1% (n = 61) received NOACs (Table 1). Both groups were predominately made up of men. In patients with NOACs, most of them were given rivaroxaban (n = 58, 95.1%) and two patients were administered dabigatran while one patient was given apixaban. Patients receiving NOACs were generally younger than those receiving VKAs. More than half of enrolled patients were diagnosed with ‘others’ diseases—hypertrophic cardiomyopathy, peripartum cardiomyopathy, myocarditis, arrhythmogenic right ventricular cardiomyopathy, hypertensive heart disease, noncompaction of ventricular myocardium, and other cardiovascular diseases, and approximately 20% of patients had ischemic cardiomyopathy (ICM) and dilated cardiomyopathy (DCM) respectively, while 55.8% of the patients with ICM experienced an acute MI with or without ventricular aneurysm. The majority of patients in our study were first diagnosed with VMT using routine echocardiography (n = 178, 90.8%), whereas the remaining patients with minor thrombus or apex thrombus were found by contrast echocardiography (n = 2, 1.0%), CT with a delayed phase scan (n = 10, 6.1%), and late gadolinium enhancement CMR imaging (n = 6, 3.1%). The median baseline LVEF was 31.5% and 83 out of 196 patients (42.3%) had LVEF <30% (8.6% with LVEF <20%, 33.7% with LVEF 20% to 30%). The international normalized ratio (INR) data for 121 (61.7%) patients were available during the follow-up, while 46 out of 121 (38.0%) patients were with a time in the therapeutic range >60%. Moreover, we also compared the characteristics of included and excluded patients in the study and summarized the prognosis of patients who were not treated with oral anticoagulation or had no imaging follow-up (Details were shown in** Supplementary Tables 1,2**).

**Table 1.** **Baseline characteristics of patients with VMT [N (%)]**.

		Total	NOACs	VKAs	*p* value
		(N = 196)	(N = 61)	(N = 135)	
Age, y [Median (IQR)]		49.0 (34.0, 58.0)	41.0 (27.0, 56.0)	50.0 (37.0, 59.0)	0.040
Male		151 (77.0)	43 (70.5)	108 (80.0)	0.200
BMI, kg/$m^{2}$ [Mean ± SD]		24.4 ± 3.9	24.6 ± 4.3	24.3 ± 3.7	0.539
Presenting diagnosis					0.150
	ICM	43 (21.9)	10 (16.4)	33 (24.4)	-
	DCM	39 (19.9)	9 (14.8)	30 (22.2)	-
	${\mathrm{Others}}^{†}$	114 (58.2)	42 (68.9)	72 (53.3)	-
Prior medical history					
	Coronary artery diseases	87 (44.4)	21 (34.4)	66 (48.9)	0.083
	Atrial fibrillation	20 (10.2)	4 (6.6)	16 (11.9)	0.316
	Heart failure	118 (60.2)	37 (60.7)	81 (60.0)	1.000
	Hypertension	56 (28.6)	17 (27.9)	39 (28.9)	1.000
	Diabetes	31 (15.8)	7 (11.5)	24 (17.8)	0.298
	Hyperlipidemia	81 (41.3)	20 (32.8)	61 (45.2)	0.140
	Embolism	48 (24.5)	17 (27.9)	31 (23.0)	0.575
	Chronic kidney diseases	9 (4.6)	1 (1.6)	8 (5.9)	0.278
	Gastrointestinal bleeding	5 (2.6)	1 (1.6)	4 (3.0)	1.000
Current smoker		102 (52.0)	30 (49.2)	72 (53.3)	0.701
Excessive alcohol ${\mathrm{consumption}}^{§}$		45 (23.0)	17 (27.9)	28 (20.7)	0.360
Location of ventricular thrombi					0.002
	Left ventricular	169 (86.2)	45 (73.8)	124 (91.9)	-
	Right ventricular	19 (9.7)	10 (16.4)	9 (6.7)	-
	Biventricular	8 (4.1)	6 (9.8)	2 (1.5)	-
Number of ventricular thrombi					0.445
	1	176 (89.8)	53 (86.9)	123 (91.1)	-
	≥2	20 (10.2)	8 (13.1)	12 (8.9)	-
Size of ventricular thrombi, mm [Median (IQR)]					
	Diameter	22.0(14.5, 30.0)	22.0 (16.0, 29.5)	22.0 (14.0, 30.2)	0.776
	Thickness	15.0 (11.0, 21.0)	16.0 (13.0, 22.0)	15.0 (10.0, 21.0)	0.305
	Width	26.0 (11.5, 44.5)	17.5 (13.2, 21.7)	42.0 (13.0, 47.0)	0.245
LVEF, % [Median (IQR)]		31.5 (25.0, 42.2)	31.0 (25.0, 45.0)	32.0 (24.0, 41.5)	0.410
D-Dimer, ug/mL [Median (IQR)]		1.35 (0.46, 2.62)	1.39 (0.47, 2.74)	1.29 (0.47, 2.61)	0.793
Combined medications					
	Parenteral anticoagulants	123 (62.8)	25 (41.0)	98 (72.6)	0.001
	Antiplatelet therapy	66 (33.7)	16 (26.2)	50 (37.0)	0.187

^†^Other diagnoses included hypertrophic cardiomyopathy, peripartum cardiomyopathy, myocarditis, arrhythmogenic right ventricular cardiomyopathy, hypertensive heart disease, and noncompaction of ventricular myocardium. ^§^Excessive alcohol consumption: >40 grams per day for women and >80 grams per day for men, lasting more than 5 years. **Abbreviations**: VMT, ventricular mural thrombus; SD, standard deviation; IQR, interquartile range; NOACs, non-vitamin K antagonist oral anticoagulants; VKAs, vitamin K antagonists; BMI, body mass index; ICM, ischemic cardiomyopathy; DCM, dilated cardiomyopathy; LVEF, left ventricular ejection fraction.

### 3.2 Primary Outcome 

A total of 40 patients (65.5%) in NOACs use were successful in resolving their thrombi at 3 months as opposed to 57 patients (42.2%) in the VKAs group (*p* = 0.004). The median time of thrombus resolved or unresolved was non-significant between the two anticoagulants (*p* = 0.921, *p* = 0.985, respectively; Table 2). When assessing the relation between thrombus resolution rates and the baseline features of patients, we conducted a Logistic regression. In the univariable analysis, patients who received NOACs had a greater risk to have the thrombus resolved than those who were in the VKAs group (OR 2.61, 95% CI 1.39 to 4.89, *p* = 0.003). The medical history of HF in patients with VMT was associated with a close to two-fold increased resolution rate compared to VMT patients without prior HF (OR 2.10, 95% CI 1.17 to 3.77, *p* = 0.013). Likewise, patients with a lower LVEF had a higher likelihood of achieving thrombus resolution (OR 0.36, 95% CI 0.20 to 0.65, *p* = 0.001) (Table 3).

**Table 2.** **Outcomes of VMT patients within 3 months follow-up [N (%)]**.

		Total	NOACs	VKAs	*p* value*
		(N = 196)	(N = 61)	(N = 135)	
Primary outcome					
	Thrombus resolution	97 (49.5)	40 (65.6)	57 (42.2)	0.004
	Time of thrombus resolved, d [Median (IQR)]	41 (30, 60)	40 (33, 51)	43 (29, 67)	0.921
	Time of thrombus unresolved, d [Median (IQR)]	48 (32, 58)	41 (30, 60)	48 (33, 57)	0.985
Secondary outcome					
	Bleeding	10 (5.1)	1 (1.6)	9 (6.7)	0.258
Thromboembolism		1 (0.5)	0 (0.0)	1 (0.7)	1.000
All-cause death		3 (1.5)	0 (0.0)	3 (2.2)	0.586

*Calculated by Fisher’s exact test. **Abbreviations**: VMT, ventricular mural thrombus; IQR, interquartile range; NOACs, non-vitamin K antagonist oral anticoagulants; VKAs, vitamin K antagonists.

**Table 3.** **Main results of univariable Logistic regression analysis**.

Variable		OR (95% CI)	*p* value
Treatments			
	NOACs vs VKAs	2.61 (1.39, 4.89)	0.003
Demography			
	Age	0.66 (0.38, 1.16)	0.152
	Male (vs Female)	1.03 (0.53, 2.01)	0.628
	BMI	1.28 (0.73, 2.24)	0.392
Presenting diagnosis			0.206
	DCM (vs ICM)	0.72 (0.30, 1.73)	0.462
	${\mathrm{Others}}^{†}$ (vs ICM)	1.37 (0.68, 2.77)	0.379
Medical history			
	Coronary artery diseases	0.66 (0.37, 1.16)	0.147
	Atrial fibrillation	1.02 (0.41, 2.58)	0.962
	Heart failure	2.10 (1.17, 3.77)	0.013
	Hypertension	0.84 (0.45, 1.57)	0.588
	Diabetes	0.95 (0.44, 2.04)	0.894
	Hyperlipidemia	0.84 (0.47, 1.48)	0.545
	Embolism	1.03 (0.54, 1.97)	0.935
	Chronic kidney diseases	0.81 (0.21, 3.11)	0.757
	Gastrointestinal bleeding	0.25 (0.03, 2.25)	0.215
Current smoker		0.82 (0.47, 1.43)	0.478
Excessive alcohol ${\mathrm{consumption}}^{§}$		0.77 (0.39, 1.50)	0.441
Location of ventricular thrombus			0.617
	Right ventricular (vs left ventricular)	0.74 (0.28, 1.92)	0.531
	Biventricular (vs left ventricular)	1.69 (0.39, 7.28)	0.484
Number of ventricular thrombus			
	≥2 (vs 1)	1.28 (0.51, 3.24)	0.604
LVEF		0.36 (0.20, 0.65)	0.001
D-Dimer		0.83 (0.42, 1.65)	0.601
Combined medications			
	Parenteral anticoagulants	0.71 (0.40, 1.27)	0.253
	Antiplatelet therapy	0.65 (0.36, 1.18)	0.160

^†^Other diagnoses included hypertrophic cardiomyopathy, peripartum cardiomyopathy, myocarditis, arrhythmogenic right ventricular cardiomyopathy, hypertensive heart disease, and noncompaction of ventricular myocardium. ^§^Excessive alcohol consumption: >40 grams per day for women and >80 grams per day for men, lasting more than 5 years. **Abbreviations**: NOACs, non-vitamin K antagonist oral anticoagulants; VKAs, vitamin K antagonists; BMI, body mass index; ICM, ischemic cardiomyopathy; DCM, dilated cardiomyopathy; LVEF, left ventricular ejection fraction; OR, odds ratio, CI, confidence interval.

**Fig. 2.**
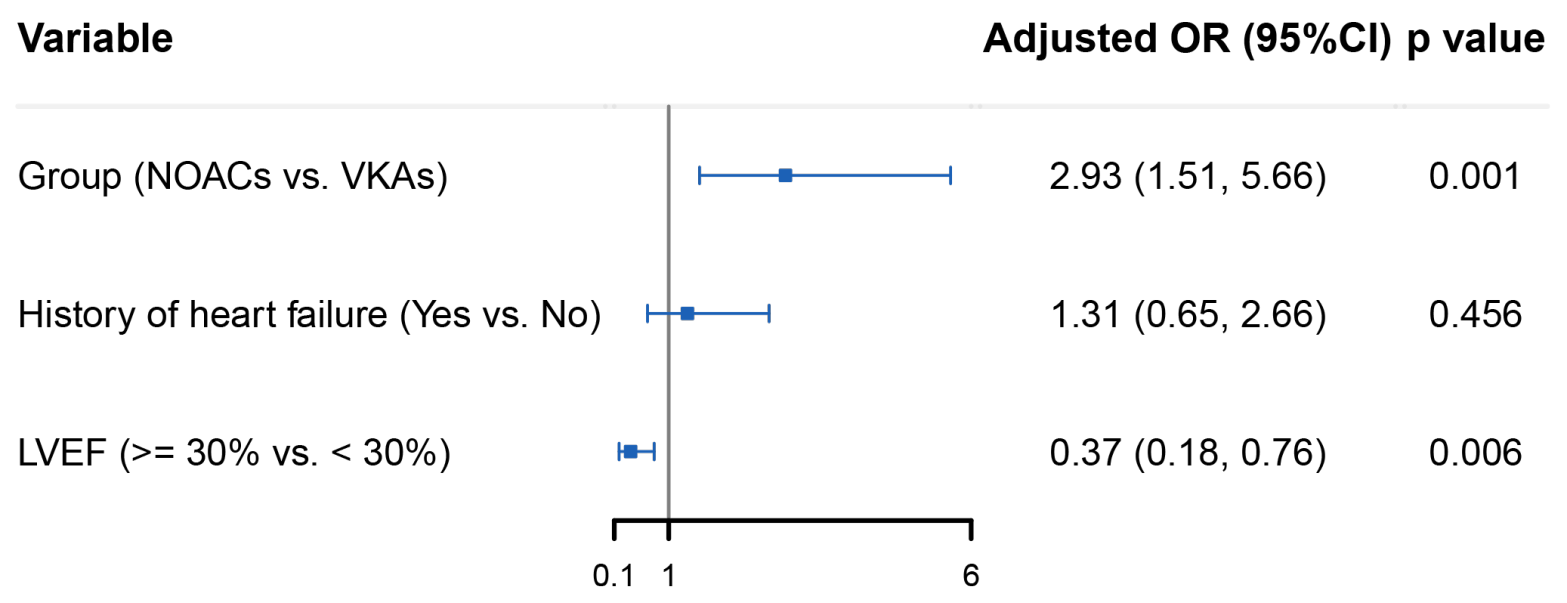
**Forest plot of multivariable analysis based on the statistically significant predictors in the univariable analysis**. Error bars represent 95% CI. NOACs, non-vitamin K antagonist oral anticoagulants; VKAs, vitamin K antagonists; OR, odds ratio; CI, confidence interval; LVEF, left ventricular ejection fraction.

And adjusting variables that were identified in the univariate analysis to be statistically significant, NOACs remained a favorable resolution of thrombus versus VKAs (OR 2.93, 95% CI 1.51 to 5.66, *p* = 0.001). In multivariable Logistic regression, patients with LVEF under 30% experienced a greater thrombus resolution than those with LVEF over 30% (OR 0.37, 95% CI 0.18 to 0.76, *p* = 0.006) (Fig. 2).

### 3.3 Secondary Outcomes 

Within a 3-month follow-up, a total of ten (5.1%) patients had minor bleeding events—one patient (1.6%) was in the NOACs group while nine (6.7%) patients were in the VKAs group (Table 2). No significant difference was observed in the bleeding rate among NOACs and VKAs group (*p* = 0.258). In the VKAs group, there was one patient (0.7%) who experienced lower extremity deep vein thrombosis. As a consequence of serious multiple organ dysfunction, progressive HF decompensation, or catastrophic infection illnesses, three patients (2.2%) died during their initial hospitalization while no patients died in the NOACs group.

### 3.4 Further Analysis

**Fig. 3.**
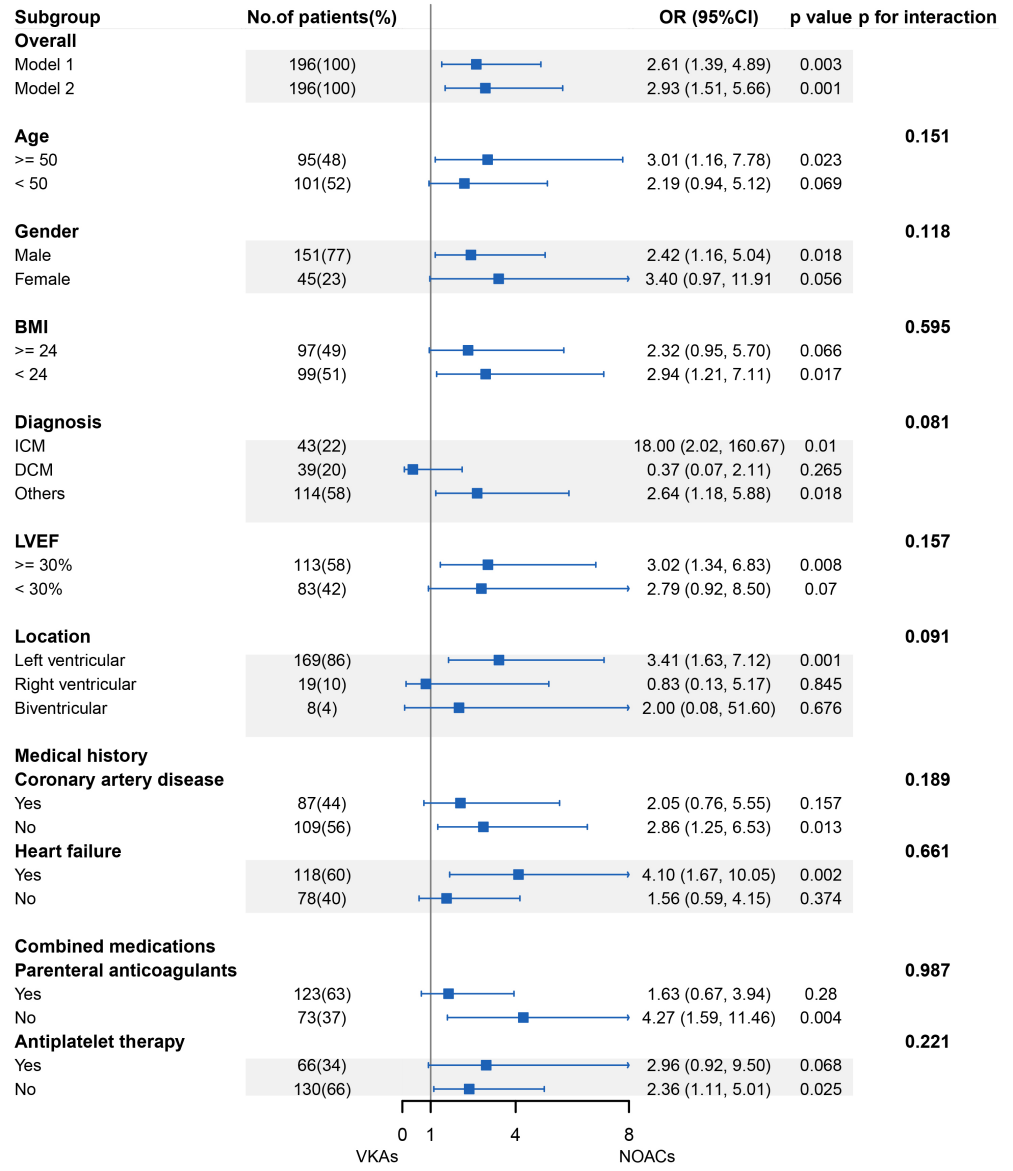
**Forest plot of subgroup analysis**. The following adjustments were performed in regression models: model 1, crude model; model 2: model 1 + history of heart failure and LVEF. Error bars represent 95% CI. NOACs, non-vitamin K antagonist oral anticoagulants; VKAs, vitamin K antagonists; OR, odds ratio; CI, confidence interval; BMI, body mass index; ICM, ischemic cardiomyopathy; DCM, dilated cardiomyopathy; Others, included hypertrophic cardiomyopathy, peripartum cardiomyopathy, myocarditis, arrhythmogenic right ventricular cardiomyopathy, hypertensive heart disease, and noncompaction of ventricular myocardium; LVEF, left ventricular ejection fraction.

To determine how confounding factors influenced thrombus resolution, two models were taken into account. In the crude model, the result echoed that of univariable analysis (OR 2.61 95% CI 1.39 to 4.89, *p* = 0.003). The statistical significance was maintained in model 2 when the medical history of HF and LVEF levels were included (OR 2.93, 95% CI 1.51 to 5.66, *p* = 0.001) (Fig. 3).

According to the subgroup study, patients over 50 years old benefit more from NOAC anticoagulation than those using VKAs (OR 3.01, 95% CI 1.16 to 7.78, *p* = 0.023), and the interaction was not significant not only across age groups (*p* = 0.171) but also between age and anticoagulation treatment (*p* = 0.627). Compared with VKAs, males using NOACs might have superior resolution compared to VKAs (OR 2.42, 95% CI 1.16 to 5.04, *p* = 0.018), whereas no significance was observed in females (OR 3.40, 95% CI 0.97 to 11.91, *p* = 0.056). Patients who had a medical history of HF experienced a greater efficacy in NOACs use than VKAs (OR 4.10, 95% CI 1.67 to 10.05, *p* = 0.002), while those without a history of coronary artery diseases (CAD) had a similar outcome (OR 2.86, 95% CI 1.25 to 6.53, *p* = 0.013). Moreover, we conducted an additional subgroup analysis of patients with left VMT alone and there was no difference in the baseline characteristics between the NOACs group and the VKAs group. And the outcome in univariate and multivariate logistic regression remained consistent with that of all VMT patients (**Supplementary Table 3**). Patients with left VMT alone in the NOACs group experienced a higher resolution of thrombus than those in the VKAs group (OR 3.41, 95% CI 1.63 to 7.12, *p* = 0.001; adjusted OR 3.79, 95% CI 1.76 to 8.19, *p *< 0.001). However, no significant differences were shown in patients with right ventricular or biventricular (OR 0.83, 95% CI 0.13 to 5.17, *p* = 0.845; OR 2.00, 95% CI 0.08 to 51.60, *p* = 0.676; separately). No significant interactions were found in the subgroups of age, gender, BMI, presenting diagnosis, medical history of CAD and HF, LVEF levels, location of thrombi, parenteral anticoagulants, and antiplatelet therapy (Fig. 3).

**Fig. 4.**
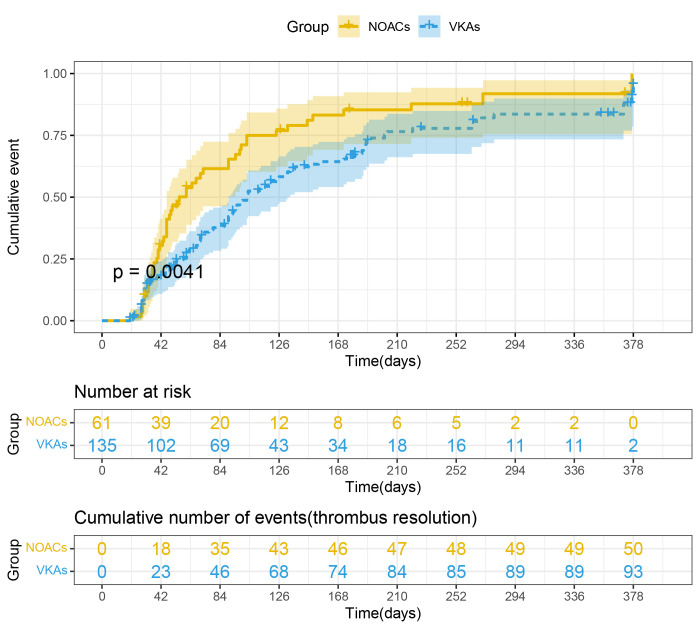
**Cumulative event probability curve for ventricular mural thrombus resolution of NOACs and VKAs within one-year follow-up**. Kaplan-Meier method was used to calculate the cumulative event probability of two oral anticoagulants. Log-rank test was used to compare the cumulative event among groups (*p* = 0.0041). NOACs, non-vitamin K antagonist oral anticoagulants; VKAs, vitamin K antagonists.

In addition, using cumulative event probability curves obtained from Kaplan-Meier estimates at one-year follow-up, we evaluated the rate of thrombus resolution between the NOACs group and the VKAs group, and the results indicated that patients receiving NOACs had higher rates of resolution than those receiving VKAs (Log-rank test, *p* = 0.0041; Fig. 4). The same analysis was conducted for patients with different levels of LVEF at baseline, which showed that patients in the group with lower LVEF had a higher rate of thrombus resolution than those with LVEF over 30% during the follow-up period (Log-rank test, *p* = 0.0015; **Supplementary Fig. 1**). And considering the LVEF as a continuous variable, we performed a restricted cubic spline curve which showed a linear relationship between the LVEF level and the thrombus resolution (*p* for linearity > 0.05; **Supplementary Fig. 2**). A lower LVEF level was significantly associated with an increased rate of thrombus resolution (*p* = 0.0030).

## 4. Discussion 

In this retrospective observational study, NOACs were shown to be significantly associated with greater resolution of VMT than VKAs in the early period of observation, with or without adjustment, and patients with a medical history of HF or LVEF <30% had a greater thrombus resolution.

### 4.1 Comparison of NOACs versus VKAs on Efficacy and Safety in Patients with VMT

In aspects of our key findings, patients diagnosed with VMT might benefit better from NOACs as a therapy option than of VKAs, which were in line with multiple additional studies [19, 20, 21, 22, 23, 24, 25, 26, 27, 28, 29, 30, 31, 32, 33, 34, 35] (**Supplementary Table 4**). Albabtain *et al*. 2021 [22] found that in the warfarin group 68.6% of patients, and in the rivaroxaban group 71.4 % of patients, respectively, obtained thrombus resolution. The median time to resolution was shorter in the rivaroxaban group of patients, which was comparable to our study [22]. Several studies found that NOACs and VKAs had comparable efficacy and safety in the treatment of patients with left VMT [21, 22, 23, 24, 25, 26]. Willeford *et al*. [21] analyzed that in either the unadjusted or the adjusted analysis, there was no noticeable difference between the NOACs and VKAs groups for the effectiveness or safety outcome. Herald *et al*. 2022 [36] supported that the NOACs treatment for left VMT could be as safe and effective as the warfarin treatment in the diverse population-based cohort of patients. The study especially focused on the safety outcome and it indicated that NOACs use was associated with a lower risk of bleeding without adding risks of embolism events [36]. Another meta-analysis also reported that patients with NOACs were less likely to experience major bleeding [37]. Chen *et al*. [38] included a total of thirteen retrospective studies with 2467 patients (NOACs = 489 vs warfarin = 1539), in terms of stroke events or clinically related bleeding events, NOACs had a lower risk than warfarin though no significant difference was observed in the resolution rate or bleeding events. And whether NOACs brought benefits or hazards in stroke or systemic embolism events was unknown. One study in 2021 included eighty-seven patients with left VMT, in the univariate logistic regression analysis, the NOACs group had a lower incidence of 66% in stroke or systemic embolism than the VKAs groups when antiplatelets were controlled [39]. Otherwise, a meta-analysis provided conflicting results that the incidence of systemic embolism in the NOACs group was 1.86 times higher than that in the VKAs group [40]. Also, Robinson *et al*. [41] discovered that the rate of systemic embolism was 2.71 times higher in the NOACs group than in the VKAs group.

### 4.2 Factors Related to Thrombus Resolution

Anticoagulants including NOACs and VKAs, which are extensively prescribed for the prevention and treatment of venous thromboembolism or stroke events, primarily act on clotting factors to prevent blood coagulation and block thrombosis [42, 43]. From the study, most of the baseline characteristics, including demographics, presenting diagnosis, medical history, thrombi features, and agent combinations, showed no correlation with the thrombus resolution. Interestingly, the rate of VMT resolution was found to be correlated with both the history of HF and lower baseline LVEF values (either being the continuous variable or the binary variable according to a cutoff of <30%). The findings could be explained for two reasons. On the one hand, owing to the pathological conditions (e.g., blood stasis and hypercoagulability) that were associated with activation of the anticoagulation and fibrinolysis system, patients who were hospitalized with severe cardiac dysfunction and experienced reduced LVEF at baseline were more likely to develop a new onset VMT, which indicated that the thrombus among these patients was easier to resolve than those calcified ones [44]. On other hand, the standardized treatment for HF may have an effect on thrombus resolution, by improving cardiac function [45]. It is possible that some of the effects of angiotensin-converting enzyme inhibitors may be mediated by beneficial effects on platelets such as reducing fibrinogen levels and improving endothelial function [46], which in turn reduces thrombosis. According to a study with 100 patients with VMT, the mean LVEF was 28.5% and the results showed that the mean LVEF improved considerably more in patients with resolved thrombus than in those without [24]. Hofer *et al*. 2021 [47] also reported that the median LVEF was lower in the group of resolved thrombus and that more than half of patients with thrombus resolution had LVEFs below 50%. In the current study, patients had a median LVEF of 31.5% and received systemic treatment during hospitalization. We might assume that patients with reduced LVEF at admission had better cardiac function under medication management, as proven by prescriptions, which increased the likelihood of thrombus resolution, though patient compliance after discharge was undetermined.

Considering the influence of these factors related to thrombus resolution, we performed subgroup analyses of different models and other potential factors. Patients treated with NOACs had a resolution rate that was over twice as high as those who administered VKAs in these models which created potential confounders. And in patients with a medical history of HF, NOACs showed a better rate of resolution than VKAs. Furthermore, our study indicated that patients >50 years old had a greater resolution in the NOACs group and the reason can be accounted for that the older patients are, the poorer adherence to VKAs they have, demonstrating that patients who are less likely to monitor their INR may use NOACs if they have no contraindications, particularly during the pandemic. Despite the fact that patients with ICM in the NOACs group had a higher likelihood of having the thrombus resolved than those in the VKAs group, the result would still be considered seriously because the confidence interval was too wide. Additionally, it remained unknown for the use of NOACs in patients with right VMT or biventricular thrombus, especially in those with thrombophilia. European Society of Cardiology guidelines recommended that the first-line anticoagulant treatment for people with antiphospholipid antibody syndrome should be warfarin rather than a NOAC [48, 49].

### 4.3 Limitations

First, given the observational study’s intrinsic limits and the small sample size, the externality of the result is further constrained. Additionally, it is difficult to determine the adherence of patients prescribed warfarin due to restrictions to INR measurements, and the net outcome of warfarin is still unclear in the current study. Second, we mostly relied on transthoracic echocardiography, which may have missed minor thrombi during the follow-up period since CMR or contrast echocardiography is the gold standard for detecting VMT.

More randomized controlled trials are required to assess the efficacy and hard outcomes in the comparison of NOACs versus VKAs and we hope those upcoming results from large trials will provide cheerful and reliable evidence on this topic (NCT03764241 [50], NCT 03415386, NCT03232398, NCT02982590, NCT04970576, ChiCTR2100048098). The evidence-based VMT guideline is critical for regulating clinician practice and guaranteeing consistency of treatment across specific doctors.

## 5. Conclusions

In this single-center retrospective cohort study, patients with a medical history of HF or LVEF <30% experienced a higher thrombus resolution. Additionally, the resolution of VMT with NOACs treatment was significantly greater than that with VKAs therapy at 3 months, with or without adjusting for baseline variables. Randomized controlled trials with long-term follow-up are required to properly evaluate the effectiveness of therapies, with the target of ultimately improving the outcome of patients with VMT.

## Data Availability

The data used to support the findings of this study are available from the corresponding author upon request.

## Abbreviations

VMT, ventricular mural thrombus; NOACs, non-vitamin K antagonist oral anticoagulants; VKAs, vitamin K antagonists; BMI, body mass index; SD, standard deviation; IQR, interquartile range; OR, odds ratio; CT, computer tomography; CMR, cardiac magnetic resonance; HF, heart failure; MI, myocardial infarction; CAD, coronary artery diseases; ICM, ischemic cardiomyopathy; DCM, dilated cardiomyopathy; LVEF, left ventricular ejection fraction; INR, international normalized ratio; ISTH, International Society on Thrombosis and Haemostasis.

## Supplementary Material

Supplementary material associated with this article can be found, in the online version, at https://doi.org/10.31083/j.rcm2403074.

## References

[1] Gianstefani S, Douiri A, Delithanasis I, Rogers T, Sen A, Kalra S (2014). Incidence and Predictors of Early Left Ventricular Thrombus after ST-Elevation Myocardial Infarction in the Contemporary Era of Primary Percutaneous Coronary Intervention. *The American Journal of Cardiology*.

[2] Massussi M, Scotti A, Lip GYH, Proietti R (2021). Left ventricular thrombosis: new perspectives on an old problem. *European Heart Journal - Cardiovascular Pharmacotherapy*.

[3] Hudec S, Hutyra M, Precek J, Latal J, Nykl R, Spacek M (2020). Acute myocardial infarction, intraventricular thrombus and risk of systemic embolism. *Biomedical Papers*.

[4] O’Gara PT, Kushner FG, Ascheim DD, Casey DE, Chung MK, de Lemos JA (2013). 2013 ACCF/AHA Guideline for the Management of ST-Elevation Myocardial Infarction. A Report of the American College of Cardiology Foundation/American Heart Association Task Force on Practice Guidelines. *Journal of the American College of Cardiology*.

[5] Chinese Society of Cardiology of Chinese Medical Association, Editorial Board of Chinese Journal of Cardiology (2019). Guidelines for the diagnosis and treatment of acute myocardial infarction in patients presenting with ST-segment elevation. *Chinese Journal of Cardiology*.

[6] Orenes-Piñero E, Esteve-Pastor MA, Valdés M, Lip GYH, Marín F (2017). Efficacy of non-vitamin-K antagonist oral anticoagulants for intracardiac thrombi resolution in nonvalvular atrial fibrillation. *Drug Discovery Today*.

[7] Ibanez B, James S, Agewall S, Antunes MJ, Bucciarelli-Ducci C, Bueno H (2018). 2017 ESC Guidelines for the management of acute myocardial infarction in patients presenting with ST-segment elevation: The Task Force for the management of acute myocardial infarction in patients presenting with ST-segment elevation of the European Society of Cardiology (ESC). *European Heart Journal*.

[8] Kleindorfer DO, Towfighi A, Chaturvedi S, Cockroft KM, Gutierrez J, Lombardi-Hill D (2021). 2021 Guideline for the Prevention of Stroke in Patients with Stroke and Transient Ischemic Attack: a Guideline from the American Heart Association/American Stroke Association. *Stroke*.

[9] Alcalai R, Butnaru A, Moravsky G, Yagel O, Rashad R, Ibrahimli M (2021). Apixaban versus Warfarin in Patients with Left Ventricular Thrombus, A Prospective Multicenter Randomized Clinical Trial. *European Heart Journal - Cardiovascular Pharmacotherapy*.

[10] Abdelnabi M, Saleh Y, Fareed A, Nossikof A, Wang L, Morsi M (2021). Comparative Study of Oral Anticoagulation in Left Ventricular Thrombi (no-LVT Trial). *Journal of the American College of Cardiology*.

[11] Fleddermann AM, Hayes CH, Magalski A, Main ML (2019). Efficacy of Direct Acting Oral Anticoagulants in Treatment of Left Ventricular Thrombus. *The American Journal of Cardiology*.

[12] Verma B, Singh A, Kumar M (2019). Use of dabigatran for treatment of left ventricular thrombus: a tertiary care center experience. *Journal of Family Medicine and Primary Care*.

[13] Huang L, Tan Y, Pan Y (2022). Systematic review of efficacy of direct oral anticoagulants and vitamin K antagonists in left ventricular thrombus. *ESC Heart Failure*.

[14] Salah HM, Goel A, Saluja P, Voruganti D, Al’Aref SJ, Paydak H (2021). Direct Oral Anticoagulants Versus Warfarin in Left Ventricular Thrombus. *American Journal of Therapeutics*.

[15] Chaosuwannakit N, Makarawate P (2021). Left Ventricular Thrombi: Insights from Cardiac Magnetic Resonance Imaging. *Tomography*.

[16] Schulman S, Kearon C (2005). Definition of major bleeding in clinical investigations of antihemostatic medicinal products in non-surgical patients. *Journal of Thrombosis and Haemostasis*.

[17] Kaatz S, Ahmad D, Spyropoulos AC, Schulman S (2015). Definition of clinically relevant non-major bleeding in studies of anticoagulants in atrial fibrillation and venous thromboembolic disease in non-surgical patients: communication from the SSC of the ISTH. *Journal of Thrombosis and Haemostasis*.

[18] Cumpston M, Li T, Page MJ, Chandler J, Welch VA, Higgins JP (2019). Updated guidance for trusted systematic reviews: a new edition of the Cochrane Handbook for Systematic Reviews of Interventions. *Cochrane Database of Systematic Reviews*.

[19] Iskaros O, Marsh K, Papadopoulos J, Manmadhan A, Ahuja T (2021). Evaluation of Direct Oral Anticoagulants Versus Warfarin for Intracardiac Thromboses. *Journal of Cardiovascular Pharmacology*.

[20] Jones DA, Wright P, Alizadeh MA, Fhadil S, Rathod KS, Guttmann O (2021). The Use of Novel Oral Anti-Coagulant’s (NOAC) compared to Vitamin K Antagonists (Warfarin) in patients with Left Ventricular thrombus after Acute Myocardial Infarction (AMI). *European Heart Journal - Cardiovascular Pharmacotherapy*.

[21] Willeford A, Zhu W, Stevens C, Thomas IC (2021). Direct Oral Anticoagulants Versus Warfarin in the Treatment of Left Ventricular Thrombus. *Annals of Pharmacotherapy*.

[22] Albabtain MA, Alhebaishi Y, Al-Yafi O, Kheirallah H, Othman A, Alghosoon H (2021). Rivaroxaban versus warfarin for the management of left ventricle thrombus. *The Egyptian Heart Journal*.

[23] Mihm AE, Hicklin HE, Cunha AL, Nisly SA, Davis KA (2021). Direct oral anticoagulants versus warfarin for the treatment of left ventricular thrombosis. *Internal and Emergency Medicine*.

[24] Varwani M H, Shah J, Ngunga M, Jeilan M (2021). Treatment and outcomes in patients with left ventricular thrombus - experiences from the Aga Khan University Hospital, Nairobi - Kenya. *Pan African Medical Journal*.

[25] Cochran JM, Jia X, Kaczmarek J, Staggers KA, Rifai MA, Hamzeh IR (2021). Direct Oral Anticoagulants in the Treatment of Left Ventricular Thrombus: a Retrospective, Multicenter Study and Meta-Analysis of Existing Data. *Journal of Cardiovascular Pharmacology and Therapeutics*.

[26] Alcalai R, Rashad R, Butnaru A, Moravsky G, Leibowitz D (2020). Apixaban versus Warfarin in Patients with Left Ventricular (LV) Thrombus, a prospective randomized trial. *European Heart Journal*.

[27] Zhou K, Zhang X, Xiao Y, Li D, Song G (2021). Effectiveness and safety of direct-acting oral anticoagulants compared to vitamin K antagonists in patients with left ventricular thrombus: a meta-analysis. *Thrombosis Research*.

[28] Trongtorsak A, Thangjui S, Kewcharoen J, Polpichai N, Yodsuwan R, Kittipibul V (2021). Direct oral anticoagulants vs. vitamin K antagonists for left ventricular thrombus: a systematic review and meta-analysis. *Acta Cardiologica*.

[29] Gue YX, Spinthakis N, Egred M, Gorog DA, Farag M (2021). Non-Vitamin K Antagonist Oral Anticoagulants Versus Warfarin for Patients with Left Ventricular Thrombus: a Systematic Review and Meta-Analysis. *The American Journal of Cardiology*.

[30] Yilmaz H, Akkus C, Duran R, Diker S, Celik S, Duran C (2021). Neutrophil-to-lymphocyte and Platelet-to-lymphocyte Ratios in those with Pulmonary Embolism in the Course of Coronavirus Disease 2019. *Indian Journal of Critical Care Medicine*.

[31] Saleiro C, Lopes J, De Campos D, Puga L, Costa M, Gonçalves L (2021). Left Ventricular Thrombus Therapy with Direct Oral Anticoagulants Versus Vitamin K Antagonists: a Systematic Review and Meta-Analysis. *Journal of Cardiovascular Pharmacology and Therapeutics*.

[32] Kumar D, Warsha FNU, Helmstetter N, Gupta V (2021). Efficacy and safety of direct oral anticoagulants for treatment of left ventricular thrombus; a systematic review. *Acta Cardiologica*.

[33] Iqbal H, Straw S, Craven TP, Stirling K, Wheatcroft SB, Witte KK (2020). Direct oral anticoagulants compared to vitamin K antagonist for the management of left ventricular thrombus. *ESC Heart Failure*.

[34] Daher J, Da Costa A, Hilaire C, Ferreira T, Pierrard R, Guichard JB (2020). Management of Left Ventricular Thrombi with Direct Oral Anticoagulants: Retrospective Comparative Study with Vitamin K Antagonists. *Clinical Drug Investigation*.

[35] Guddeti RR, Anwar M, Walters RW, Apala D, Pajjuru V, Kousa O (2020). Treatment of Left Ventricular Thrombus with Direct Oral Anticoagulants: a Retrospective Observational Study. *The American Journal of Medicine*.

[36] Herald J, Goitia J, Duan L, Chen A, Lee M (2022). Safety and Effectiveness of Direct Oral Anticoagulants Versus Warfarin for Treating Left Ventricular Thrombus. *American Journal of Cardiovascular Drugs*.

[37] Camilli M, Lombardi M, Del Buono MG, Chiabrando JG, Vergallo R, Niccoli G (2021). Direct oral anticoagulants vs. vitamin K antagonists for the treatment of left ventricular thrombosis: a systematic review of the literature and meta-analysis. *European Heart Journal - Cardiovascular Pharmacotherapy*.

[38] Chen Y, Zhu M, Wang K, Xu Q, Ma J (2022). Direct Oral Anticoagulants Versus Vitamin K Antagonists for the Treatment of Left Ventricular Thrombus: an Updated Meta-Analysis of Cohort Studies and Randomized Controlled Trials. *Journal of Cardiovascular Pharmacology*.

[39] Xu Z, Li X, Li X, Gao Y, Mi X (2021). Direct oral anticoagulants versus vitamin K antagonists for patients with left ventricular thrombus. *Annals of Palliative Medicine*.

[40] Burmeister C, Beran A, Mhanna M, Ghazaleh S, Tomcho JC, Maqsood A (2021). Efficacy and Safety of Direct Oral Anticoagulants Versus Vitamin K Antagonists in the Treatment of Left Ventricular Thrombus. *American Journal of Therapeutics*.

[41] Robinson AA, Trankle CR, Eubanks G, Schumann C, Thompson P, Wallace RL (2020). Off-label Use of Direct Oral Anticoagulants Compared with Warfarin for Left Ventricular Thrombi. *JAMA Cardiology*.

[42] Kamel H, Healey JS (2017). Cardioembolic Stroke. *Circulation Research*.

[43] Yasuda S, Kaikita K, Akao M, Ako J, Matoba T, Nakamura M (2019). Antithrombotic Therapy for Atrial Fibrillation with Stable Coronary Disease. *New England Journal of Medicine*.

[44] Sumaya W, Storey RF (2018). The challenges of antithrombotic therapy in patients with left ventricular thrombosis. *European Heart Journal*.

[45] Blann A, Lip G, Islim I, Beevers D (1997). Evidence of platelet activation in hypertension. *Journal of Human Hypertension*.

[46] Gibbs CR, Blann AD, Watson RDS, Lip GYH (2001). Abnormalities of Hemorheological, Endothelial, and Platelet Function in Patients with Chronic Heart Failure in Sinus Rhythm: effects of angiotensin-converting enzyme inhibitor and beta-blocker therapy. *Circulation*.

[47] Hofer F, Kazem N, Schweitzer R, Horvat P, Winter MP, Koller L (2021). The prognostic impact of left ventricular thrombus resolution after acute coronary syndrome and risk modulation via antithrombotic treatment strategies. *Clinical Cardiology*.

[48] Zuily S, Cohen H, Isenberg D, Woller SC, Crowther M, Dufrost V (2020). Use of direct oral anticoagulants in patients with thrombotic antiphospholipid syndrome: Guidance from the Scientific and Standardization Committee of the International Society on Thrombosis and Haemostasis. *Journal of Thrombosis and Haemostasis*.

[49] Tektonidou MG, Andreoli L, Limper M, Amoura Z, Cervera R, Costedoat-Chalumeau N (2019). EULAR recommendations for the management of antiphospholipid syndrome in adults. *Annals of the Rheumatic Diseases*.

[50] He J, Ge H, Dong JX, Zhang W, Kong LC, Qiao ZQ (2020). Rationale and design of a prospective multi-center randomized trial of EARLY treatment by rivaroxaban versus warfarin in ST-segment elevation MYOcardial infarction with Left Ventricular Thrombus (EARLY-MYO-LVT trial). *Annals of Translational Medicine*.

