# Supplementary material for: Characteristics of Novel Anticoagulants versus Vitamin K Antagonists in the Ventricular Mural Thrombus
Source: Rev Cardiovasc Med. 2023 Mar 2;24(3):74. doi: 10.31083/j.rcm2403074 (PMC11263983; doi:10.31083/j.rcm2403074)
Supplement: Supplementary file 1 [file 2153-8174-24-3-074-s1.docx]

**Characteristics of Novel Anticoagulants versus Vitamin K antagonists in the Ventricular Mural Thrombus**

Qing Yang^1,2^, Yan Liang^1,2*^, Xin Quan^1,3^, Xinyue Lang^1,4^, Dongfang Gao^1,2^

*^1^National Clinical Research Center of Cardiovascular Diseases, Fuwai Hospital, National Center for Cardiovascular Diseases, Chinese Academy of Medical Sciences and Peking Union Medical College, Beijing 100037, China*

*^2^Emergency Center, Fuwai Hospital, National Center for Cardiovascular Diseases, Chinese Academy of Medical Sciences and Peking Union Medical College, Beijing 100037, China*

*^3^Echocardiographic Imaging Center, Fuwai Hospital, National Center for Cardiovascular Diseases, Chinese Academy of Medical Sciences and Peking Union Medical College, Beijing 100037, China.*

*^4^Medical Research & Biometrics Center, National Center for Cardiovascular Diseases, Chinese Academy of Medical Sciences, Beijing 102300, China*

**Figure and Table Legends**

**Fig.S1** Cumulative event probability curve for ventricular mural thrombus resolution of different levels of LVEF within one-year follow-up. Kaplan-Meier method was used to calculate the cumulative event probability. Log-rank test was used to compare the cumulative event among groups (P = 0.0015). LVEF: left ventricular ejection fraction.

**Fig.S2** Restricted cubic spline curve to fit the relationship between levels of LVEF and ventricular mural thrombus resolution. The model adjusted for age, sex, medical history of heart failure, and treatment group (P = 0.0030). The shaded area indicated 95% CI. LVEF: left ventricular ejection fraction; HR: hazard ratio; CI: confidence interval.

**Table S1.** Characteristics of enrolled and excluded patients with VMT [N (%)]

^†^Other diagnoses included hypertrophic cardiomyopathy, peripartum cardiomyopathy, myocarditis, arrhythmogenic right ventricular cardiomyopathy, hypertensive heart disease, and noncompaction of ventricular myocardium.

^§^Excessive alcohol consumption: >40 grams per day for women and > 80 grams per day for men, lasting more than 5 years.

**Abbreviations:** VMT: ventricular mural thrombus; N: numbers of patients; SD: standard deviation; IQR: interquartile range; NOACs: non-vitamin K antagonist oral anticoagulants; VKAs: vitamin K antagonists; BMI: body mass index; ICM: ischemic cardiomyopathy; DCM: dilated cardiomyopathy; LVEF: left ventricular ejection fraction.

**Table S2.** Main outcomes of excluded patients within 3 months follow-up [N (%)]

Abbreviations: VMT: ventricular mural thrombus; N: numbers of patients; OA: oral anticoagulation therapy.

**Table S3.** Results of Logistic regression analysis in patients with left VMT

†Other diagnoses included hypertrophic cardiomyopathy, peripartum cardiomyopathy, myocarditis, arrhythmogenic right ventricular cardiomyopathy, hypertensive heart disease, and noncompaction of ventricular myocardium.

§Excessive alcohol consumption: >40 grams per day for women and > 80 grams per day for men, lasting more than 5 years.

Abbreviations: VMT: ventricular mural thrombus; N: numbers of patients; SD: standard deviation; IQR: interquartile range; NOACs: non-vitamin K antagonist oral anticoagulants; VKAs: vitamin K antagonists; BMI: body mass index; ICM: ischemic cardiomyopathy; DCM: dilated cardiomyopathy; LVEF: left ventricular ejection fraction.

**Table S4.** Literature reviewed [N (%)]

Abbreviations: N: numbers of patients; NOACs: non-vitamin K antagonist oral anticoagulants; VKAs: vitamin K antagonists.

**Supplementary Figure**

**Fig.S1** Cumulative event probability curve for ventricular mural thrombus resolution of different levels of LVEF within one-year follow-up. Kaplan-Meier method was used to calculate the cumulative event probability. Log-rank test was used to compare the cumulative event among groups (P = 0.0015). LVEF: left ventricular ejection fraction.

**Fig.S2** Restricted cubic spline curve to fit the relationship between levels of LVEF and ventricular mural thrombus resolution. The model adjusted for age, sex, medical history of heart failure, and treatment group (P = 0.0030). The shaded area indicated 95% CI. LVEF: left ventricular ejection fraction; HR: hazard ratio; CI: confidence interval.

**Supplementary Table**

**In Table S1**, we compared the characteristics of included and excluded patients in the study, and the result showed that there were significant differences in the demography, presenting diagnosis, medical history of coronary artery diseases (CAD) and heart failure (HF), level of left ventricular ejection fraction (LVEF) and combined medications. Patients enrolled in our final analysis were younger and had a lower rate of CAD while a higher rate of HF as well as a lower LVEF, compared with those patients who were not treated with oral anticoagulation or had no imaging follow-up.

**In Table S2**, we summarized the prognosis of excluded patients as followed. In patients who had not received oral anticoagulation, 4 out of 116 patients showed positive fecal occult blood tests; no patient experienced stroke or death events in hospital. In a total of 165 patients who were lost to imaging follow-up, 12 out of 165 had bleeding events during hospitalization (one had major bleeding with intracranial bleeding and one had bruising, while the other 10 patients reported positive fecal occult blood tests), while one patient experienced a stroke event and four patients died before discharge.

**In Table S3**, we performed an additional subgroup analysis of patients with left ventricular mural thrombus (VMT) alone. The outcome in univariate and multivariate logistic regression remained consistent with that of all VMT patients. Patients with left VMT alone in the non-vitamin K antagonist oral anticoagulants (NOACs) group had a greater risk to have the thrombus resolved than those who were in the vitamin K antagonists (VKAs) group (OR 3.41, 95% CI 1.63 to 7.12, p = 0.001). And adjusting the medical history of HF and LVEF levels, the difference remained (OR 3.79, 95% CI 1.76 to 8.19, p < 0.001). In multivariable Logistic regression, patients whose LVEF < 30% might have a better resolution of thrombus than those with LVEF ≥ 30% (OR 0.39, 95% CI 0.19 to 0.84, p = 0.016).

**In Table S4**, we presented the main results of a total of 23 articles comparing NOACs with VKAs in the treatment of ventricular thrombus.

**Table S1.** Characteristics of enrolled and excluded patients with VMT [N (%)]

|  | **Total**  **(N=500)** | **Excluded**  **(N=304)** | **Enrolled**  **(N=196)** | **P value** |
| --- | --- | --- | --- | --- |
| **Age, y [Median (IQR)]** | 54.0 (43.0, 63.0) | 57.0 (46.7, 64.0) | 49.0 (34.0, 58.0) | 0.001 |
| **Male** | 410 (82.0) | 259 (85.2) | 151 (77.0) | 0.028 |
| **BMI, kg/m^2^ [Mean ± SD]** | 24.9±3.4 | 25.3±3.0 | 24.4±3.9 | 0.002 |
| **Presenting diagnosis** |  |  |  | 0.001 |
| ICM | 292 (58.4) | 249 (81.9) | 43 (21.9) | - |
| DCM | 62 (12.4) | 23 (7.6) | 39 (19.9) | - |
| Others^†^ | 146 (29.2) | 32 (10.5) | 114 (58.2) | - |
| **Prior medical history** |  |  |  |  |
| Coronary artery diseases | 333 (66.6) | 246 (80.9) | 87 (44.4) | 0.001 |
| Atrial fibrillation | 40 (8.0) | 20 (6.6) | 20 (10.2) | 0.197 |
| Heart failure | 197 (39.4) | 79 (26.0) | 118 (60.2) | 0.001 |
| Hypertension | 186 (37.2) | 130 (42.8) | 56 (28.6) | 0.002 |
| Diabetes | 108 (21.6) | 77 (25.3) | 31 (15.8) | 0.016 |
| Hyperlipidemia | 268 (53.6) | 187 (61.5) | 81 (41.3) | 0.001 |
| Embolism | 111 (22.2) | 63 (20.7) | 48 (24.5) | 0.379 |
| Chronic kidney diseases | 28 (5.6) | 19 (6.2) | 9 (4.6) | 0.556 |
| Gastrointestinal bleeding | 6 (1.2) | 1 (0.3) | 5 (2.6) | 0.071 |
| **Current smoker** | 229 (56.8) | 127 (61.4) | 102 (52.0) | 0.074 |
| **Excessive alcohol consumption**^§^ | 95 (23.6) | 50 (24.2) | 45 (23.0) | 0.869 |
| **Location of ventricular thrombi** |  |  |  | 0.011 |
| Left ventricular | 455 (91.0) | 286 (94.1) | 169 (86.2) | - |
| Right ventricular | 31 (6.2) | 12 (3.9) | 19 (9.7) | - |
| Biventricular | 14 (2.8) | 6 (2.0) | 8 (4.1) | - |
| **Number of ventricular thrombi** |  |  |  | 0.171 |
| 1 | 442 (92.1) | 266 (93.7) | 176 (89.8) | - |
| ≥2 | 38 (7.9) | 18 (6.3) | 20 (10.2) | - |
| **Size of ventricular thrombi, mm**  **[Median (IQR)]** |  |  |  |  |
| Diameter | 21.0 (14.0, 30.0) | 19.0 (14.0, 30.0) | 22.0(14.5, 30.0) | 0.582 |
| Thickness | 16.0 (11.0, 23.0) | 17.0 (11.0, 25.0) | 15.0 (11.0, 21.0) | 0.280 |
| Width | 17.0 (13.0, 38.0) | 17.0 (16.0, 29.0) | 26.0 (11.5, 44.5) | 0.732 |
| **LVEF, % [Median (IQR)]** | 38.0 (28.0, 46.0) | 40.0 (30.5, 48.0) | 31.5 (25.0, 42.2) | 0.001 |
| **D-Dimer, ug/mL**  **[Median (IQR)]** | 1.06 (0.42, 2.59) | 0.86 (0.39, 2.43) | 1.35 (0.46, 2.62) | 0.075 |
| **Combined medications** |  |  |  |  |
| Parenteral anticoagulants | 285 (57.0) | 162 (53.3) | 123 (62.8) | 0.046 |
| Antiplatelet therapy | 266 (53.2) | 200 (65.8) | 66 (33.7) | 0.001 |

^†^Other diagnoses included hypertrophic cardiomyopathy, peripartum cardiomyopathy, myocarditis, arrhythmogenic right ventricular cardiomyopathy, hypertensive heart disease, and noncompaction of ventricular myocardium.

^§^Excessive alcohol consumption: >40 grams per day for women and > 80 grams per day for men, lasting more than 5 years.

**Abbreviations:** VMT: ventricular mural thrombus; N: numbers of patients; SD: standard deviation; IQR: interquartile range; NOACs: non-vitamin K antagonist oral anticoagulants; VKAs: vitamin K antagonists; BMI: body mass index; ICM: ischemic cardiomyopathy; DCM: dilated cardiomyopathy; LVEF: left ventricular ejection fraction.

**Table S2.** Main outcomes of excluded patients within 3 months follow-up [N (%)]

|  | **No OAC (N=116)** | **No image (N=165)** |
| --- | --- | --- |
| **Bleeding** | 4 (3.4) | 12 (7.3) |
| **Thromboembolism** | 0 (0.0) | 1 (0.6) |
| **All-cause death** | 0 (0.0) | 4 (2.4) |

**Abbreviations:** VMT: ventricular mural thrombus; N: numbers of patients; OAC: oral anticoagulation therapy.

**Table S3.** Results of Logistic regression analysis in patients with left VMT

| **Variable** | **Univariate** | | **Multivariate** | |
| --- | --- | --- | --- | --- |
|  | **OR (95% CI)** | **P value** | **OR (95% CI)** | **P value** |
| **Treatments** |  |  |  |  |
| NOACs vs VKAs | 3.41 (1.63, 7.12) | <0.001 | 3.79 (1.76, 8.19) | <0.001 |
| **Demography** |  |  |  |  |
| Age | 0.77 (0.42, 1.41) | 0.398 | - | - |
| Male (vs. Female) | 1.24 (0.58, 2.65) | 0.587 | - | - |
| BMI | 1.50 (0.82, 2.75) | 0.191 | - | - |
| **Presenting diagnosis** |  |  |  |  |
| DCM (vs ICM) | 0.68 (0.28, 1.67) | 0.462 | - | - |
| Others^†^ (vs ICM) | 1.25 (0.60, 2.63) | 0.379 | - | - |
| **Prior medical history** |  |  |  |  |
| Coronary artery disease | 0.73 (0.40, 1.34) | 0.316 | - | - |
| Atrial fibrillation | 1.14 (0.44, 2.97) | 0.787 | - | - |
| Heart failure | 2.20 (1.17, 4.14) | 0.015 | 1.44 (0.67, 3.09) | 0.351 |
| Hypertension | 1.02 (0.53, 1.96) | 0.959 | - | - |
| Diabetes | 0.93 (0.42, 2.08) | 0.866 | - | - |
| Hyperlipidemia | 1.02 (0.56, 1.88) | 0.946 | - | - |
| Embolism | 1.02 (0.51, 2.04) | 0.965 | - | - |
| Chronic kidney disease | 0.80 (0.21, 3.09) | 0.746 | - | - |
| Gastrointestinal bleeding | 0.24 (0.03, 2.23) | 0.211 | - | - |
| **Current smoker** | 0.93 (0.51, 1.71) | 0.823 | - | - |
| **Excessive alcohol consumption**^§^ | 0.84 (0.42, 1.69) | 0.628 | - | - |
| **Numbers of left VMT** |  |  |  |  |
| ≥2 (vs. 1) | 0.97 (0.94, 1.00) | 0.059 | - | - |
| **LVEF** | 0.37 (0.20, 0.69) | 0.002 | 0.39 (0.19, 0.84) | 0.016 |
| **D-Dimer** | 0.90 (0.44, 1.88) | 0.788 | - | - |
| **Combined medications** |  |  |  |  |
| Parenteral anticoagulants | 0.64 (0.34, 1.22) | 0.178 | - | - |
| Antiplatelet therapy | 0.71 (0.38, 1.34) | 0.292 | - | - |

^†^Other diagnoses included hypertrophic cardiomyopathy, peripartum cardiomyopathy, myocarditis, arrhythmogenic right ventricular cardiomyopathy, hypertensive heart disease, and noncompaction of ventricular myocardium.

^§^Excessive alcohol consumption: >40 grams per day for women and > 80 grams per day for men, lasting more than 5 years.

**Abbreviations:** VMT: ventricular mural thrombus; N: numbers of patients; OR: odds ratio, CI: confidence interval; SD: standard deviation; IQR: interquartile range; NOACs: non-vitamin K antagonist oral anticoagulants; VKAs: vitamin K antagonists; BMI: body mass index; ICM: ischemic cardiomyopathy; DCM: dilated cardiomyopathy; LVEF: left ventricular ejection fraction.

**Table S4.** Literature reviewed [N (%)]

| **Study** | **Comparison** | **Sample size** | **Thrombus resolution** | **Bleeding** | **Stroke or systemic embolism** | **All-cause death** |
| --- | --- | --- | --- | --- | --- | --- |
| **Yao et al,**  **2021** | NOACs | 42 | 29(69) | 0 | - | - |
|  | VKA | 58 | 37(64) | 0 | 2(3) | - |
| **Iskaros et al,**  **2021** | NOACs | 32 | 27(84) | 2(7) | 5(17) | - |
|  | VKA | 45 | 34(75) | 2(5) | 11(26) | - |
| **Zhang et al,**  **2021** | NOACs | 33 | 26(78.8) | 0 | 1(3) | 1(3) |
|  | VKA | 31 | 23(74.2) | 1(1.6) | 4(12.9) | 4(12.9) |
| **Mihm et al,**  **2021** | NOACs | 33 | 14/24(58.3) | 5(15) | 3(9.1) | 4(12.1) |
|  | VKA | 75 | 26/40(65) | 2(2.7) | 4(5.3) | 6(8) |
| **Alcalai et al,**  **2021** | NOACs | 18 | 16(94.1) | 0 | 0 | 1(5.56) |
|  | VKA | 17 | 14(93.3) | 2(11.76) | 1(5.88) | 0 |
| **Albabtain et al, 2021** | NOACs | 28 | 20(71.4) | 2(7.14) | 2(7.14) | 2(7.14) |
|  | VKA | 35 | 24(68.6) | 1(2.86) | 1(2.86) | 3(8.57) |
| **Varwani et al, 2021** | NOACs | 58 | 22(61.1) | 3(5.2) | 1(1.7) | 0 |
|  | VKA | 34 | 16(64) | 2(5.9) | 1(2.9) | 0 |
| **Jones et al,**  **2020** | NOACs | 41 | 39(95) | 0 | 1(2.4) | 0 |
|  | VKA | 60 | 51(85) | 4(6.7) | 3(5) | 0 |
| **Daher et al,**  **2020** | NOACs | 17 | 12(71) | - | 2(12) | 0 |
|  | VKA | 42 | 30(71) | - | 4(9) | 0 |
| **Yunis et al,**  **2020** | NOACs | 64 | 64(100) | 26(40) | 3(5) | 12(19) |
|  | VKA | 200 | 194(97) | 70(35) | 16(8) | 42(21) |
| **Ali et al,**  **2020** | NOACs | 32 | 18(53) | 2(6) | - | - |
|  | VKA | 60 | 37(63) | 15(27) | 2(3) | - |
| **Iqbal et al,**  **2020** | NOACs | 22 | 13(65) | 0 | 0 | 3(14) |
|  | VKA | 62 | 42(76) | 3(5) | 2(3) | 6(10) |
| **Robinson et al,**  **2020** | NOACs | 121 | 56(46) | 8(7) | 17(14) | 15(12) |
|  | VKA | 236 | 131(55) | 19(8) | 14(6) | 33(14) |
| **Willeford et al,**  **2020** | NOACs | 22 | 13(59) | 0 | 1(4) | - |
|  | VKA | 129 | 63(49) | 8(6) | 5(4) | - |
| **Bass et al,**  **2020** | NOACs | 180 | - | 60(33) | 20(11) | - |
|  | VKA | 769 | - | 235(31) | 60(8) | - |
| **Guddeti et al,**  **2020** | NOACs | 19 | 15(80) | 1(5) | 0 | 0 |
|  | VKA | 80 | 65(81) | 4(5) | 2(2) | 0 |
| **Gama et al,**  **2019** | NOACs | 13 | 11(91) | - | - | - |
|  | VKA | 53 | 31(70) | - | - | - |
| **Yan et al,**  **2019** | NOACs | 11 | 7(64) | 0 | 0 | 0 |
|  | VKA | 37 | 19(51) | 2(5) | 3(8) | 1(3) |
| **Alizadeh et al,**  **2019** | NOACs | 38 | 28(75) | 0 | - | - |
|  | VKA | 60 | 32(53) | 3(5) | - | - |
| **McCarthy et al, 2019** | NOACs | 4 | 4(100) | - | - | - |
|  | VKA | 94 | 71(75) | - | - | - |
| **Robinson et al,**  **2018** | NOACs | 35 | - | - | 2(88) | - |
|  | VKA | 40 | - | - | 3(78) | - |
| **Chao et al,**  **2018** | NOACs | 56 | 29(52) | 5(9) | 2(4) | 0 |
|  | VKA | 70 | 22(31) | 11(16) | 12(17) | 0 |
| **Li et al,**  **2015** | NOACs | 15 | 13(87) | 0 | 1(7) | 0 |
|  | VKA | 16 | 12(75) | 0 | 1(6) | 0 |

**Abbreviations:** N: numbers of patients; NOACs: non-vitamin K antagonist oral anticoagulants; VKAs: vitamin K antagonists.
